# Oxidations of Benzhydrazide and Phenylacetic Hydrazide by Hexachloroiridate(IV): Reaction Mechanism and Structure–Reactivity Relationship

**DOI:** 10.3390/molecules25020308

**Published:** 2020-01-12

**Authors:** Xiaolai Zhang

**Affiliations:** College of Chemistry and Chemical Engineering, Shandong University, Jinan 250014, China; zhangxlai@sdu.edu.cn; Tel.: +86-0531-8839-2606

**Keywords:** benzhydrazide, phenylacetic hydrazide, hexachloroirridate(IV), oxidation, reaction mechanism, structure–reactivity relationship

## Abstract

Benz(o)hydrazide (BH) is the basic aryl hydrazide; aryl hydrazides have been pursued in the course of drug discovery. Oxidations of BH and phenylacetic hydrazide (PAH) by hexachloroiridate(IV) ([IrCl_6_]^2−^) were investigated by use of stopped-flow spectral, rapid spectral scan, RP-HPLC and NMR spectroscopic techniques. The oxidation reactions followed well-defined second-order kinetics and the observed second-order rate constant *k*′ versus pH profiles were established over a wide pH range. Product analysis revealed that BH and PAH were cleanly oxidized to benzoic acid and phenylacetic acid, respectively. A reaction mechanism was proposed, resembling those suggested previously for the oxidations of isoniazid (INH) and nicotinic hydrazide (NH) by [IrCl_6_]^2−^. Rate constants of the rate-determining steps were evaluated, confirming a huge reactivity span of the protolysis species observed previously. The enolate species of BH is extremely reactive towards reduction of [IrCl_6_]^2−^. The determined middle-ranged negative values of activation entropies together with rapid scan spectra manifest that an outer-sphere electron transfer is probably taking place in the rate-determining steps. The reactivity of neutral species of hydrazides is clearly not correlated to the corresponding p*K*_a_ values of the hydrazides. On the other hand, a linear correlation, log*k*_enolate_ = (0.16 ± 0.07)p*K*_enol_ + (6.1 ± 0.8), is found for the aryl hydrazides studied so far. The big intercept and the small slope of this correlation may pave a way for a rational design of new antioxidants based on aryl hydrazides. The present work also provides the p*K*_a_ values for BH and PAH at 25.0 °C and 1.0 M ionic strength which were not reported before.

## 1. Introduction

Hydrazides are widely employed for manufactures of polymers and glues and utilized as chemical preservers for plants in industry [1,2]. They are potent reagents for synthesis of various oxygen-, nitrogen-, and/or sulfur-containing heterocyclic rings in organic chemistry [1]. Hydrazide scaffold based clinical medicines involve isoniazid (INH), marplan, iproniazid, and indolylglyoxyl hydrazide [2] and INH has been a frontline anti-tubercular drug for a few decades [3,4,5]. Ascribed to the structural diversity and to the huge success of INH, hydrazides and their derivatives have been a base for new drug discoveries [2,6,7,8,9,10].

Mechanistically, the anti-tubercular action of INH involves its activation by enzymes generating hydrazyl free radical(s) in the activation course [11,12,13,14]. The involvement of free radicals may not be surprising since numerous oxidation reactions are taking place in biologically and/or biomedically relevant processes which involve a single electron transfer. [IrCl_6_]^2−^ is a well-known single electron oxidizing agent [15,16,17,18,19] and it has been utilized as a redox probe for acquiring chemical information of oxidative stress [20]. In a recent kinetic study of oxidations of INH and its analog nicotinic hydrazide (NH) by [IrCl_6_]^2−^ [14], it was found for the first time that the four protolysis species of INH and NH showed a huge reactivity difference (about 10^9^ times) towards reduction of the Ir(IV) complex and the enolate forms of INH and NH were extremely reactive. Inspired by this study, we carried out a similar kinetic investigation of the oxidations of benz(o)hydrazide (BH) and phenylacetic hydrazide (PAH); the structures of INH, NH, BH, and PAH are illustrated in Figure S1 in Supplementary Materials (SM). Structurally, BH is the basic aryl hydrazide and has a close proximity to INH. Indeed, BH and its derivatives have been investigated extensively towards discovery of new drugs [9,10,21,22] and catalysts [23]. The oxidation kinetics of BH by bromate in acidic media catalyzed by V(IV) and Mn(II) [24,25] and by enzymes [26,27] were reported; but in these studies, the enol/enolate form of BH was not considered.

The aims of this investigation were: (i) to characterize the oxidations of BH and PAH by the Ir(IV) complex in a wide pH range by use of stopped-flow spectral, rapid spectral scan, RP-HPLC and NMR spectroscopic techniques; (ii) to derive the reactivity of the protolysis species of the two hydrazides towards reduction of Ir(IV); (iii) to verify the exceptional high reactivity of the enolate forms of BH and PAH; (iv) to compare the kinetic data of BH with those of INH; and (v) to examine the structure–reactivity relationships.

## 2. Results

### 2.1. Spectral Analysis of Reaction Course

**Figure 1:**
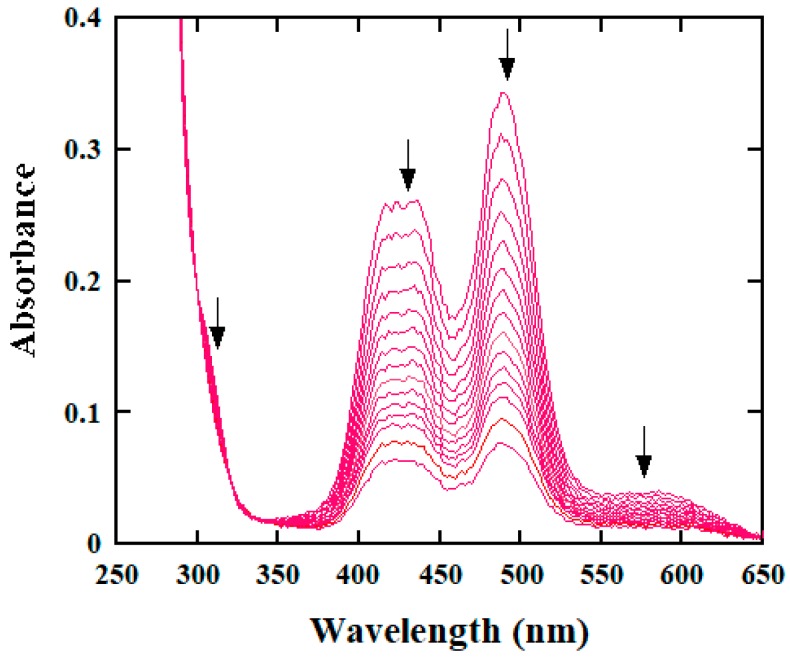
Rapid scan spectra obtained for the oxidation of benz(o)hydrazide (BH) by [IrCl_6_]^2−^ under the reaction conditions: [Ir(IV)] = 0.20 mM, [BH]_tot_ = 3.00 mM, phosphate buffer of pH 5.10, *μ* = 1.0 M and 25.0 °C. The spectra were acquired at 3, 33, 63, 92, 123, 152, 182, 212, 242, 273, 303, 332, 364, 423, and 500 milliseconds after the start of the reaction.

For probing some useful information in the reaction course of BH with [IrCl_6_]^2−^, rapid scan spectra were recorded on the stopped-flow spectrometer under a set of reaction conditions. The obtained spectra are shown in Figure 1, which are similar to those acquired previously in the oxidations of INH and hydrazines with [IrCl_6_]^2−^ [14,28]. This type of rapid scan spectra renders a few salient features [14,28]: (1) neither a substitution reaction on the Ir(IV) complex nor a strong association between the [IrCl_6_]^2−^ and BH before the rate-determining step(s) underwent since the two disappearing bands around 428 and 488 nm which originate from [IrCl_6_]^2−^ showed no shifts and no new absorption bands emerged. (2) The kinetic traces could be well described by Equation (1) under the pseudo first-order reaction conditions,

*A*_t_ = (*A*_0_ − *A*_∞_)exp(−*k*_obsd_*t*) + *A*_∞_ (1)

where *k*_obsd_ stands for pseudo first-order rate constant, and *A*_t_, *A*_0_, and *A*_∞_ represent the absorbances at time *t*, zero, and infinity, respectively. Figure S2 in SM shows good fits of the kinetic traces acquired at 428 and 488 nm. (3) The values of *k*_obsd_ obtained at the two wavelengths are identical within experimental errors. These features endow that the oxidation reaction is indeed first-order in [IrCl_6_^2−^]. The same features were also observed for the oxidation of PAH by [IrCl_6_]^2−^.

### 2.2. Empirical Rate Law and Kinetic Data Collection

**Figure 2:**
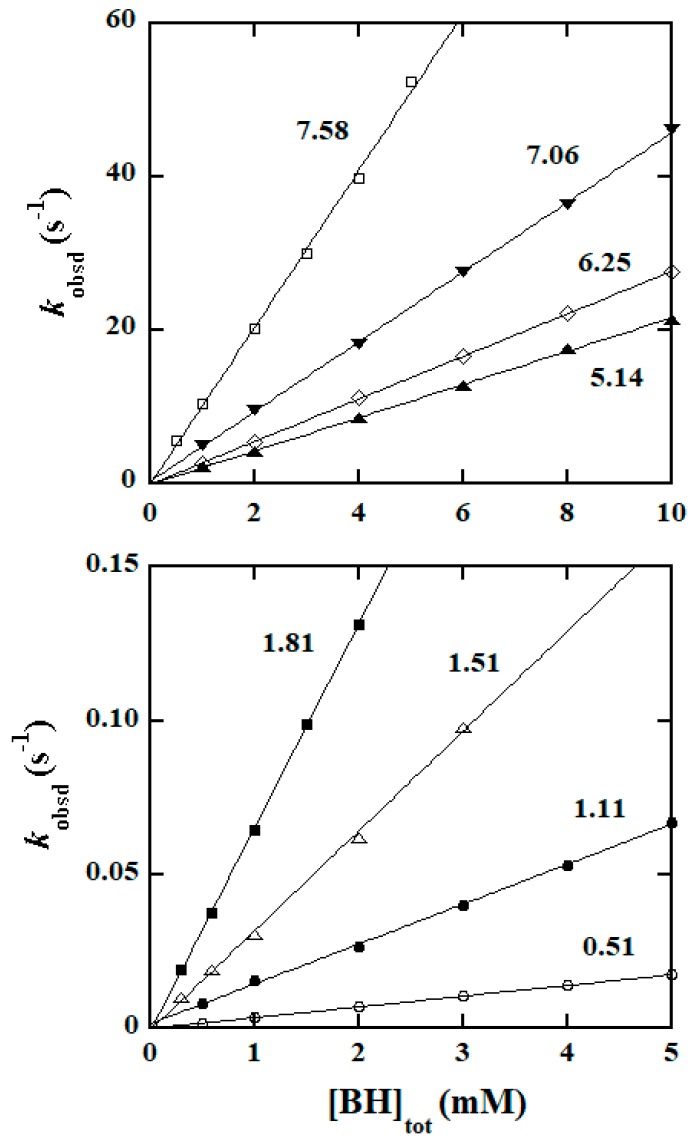
Plots of *k*_obsd_ versus [BH]_tot_ at 25.0 °C and different pHs with the numbers around the lines indicating the pH values.

**Figure 3:**
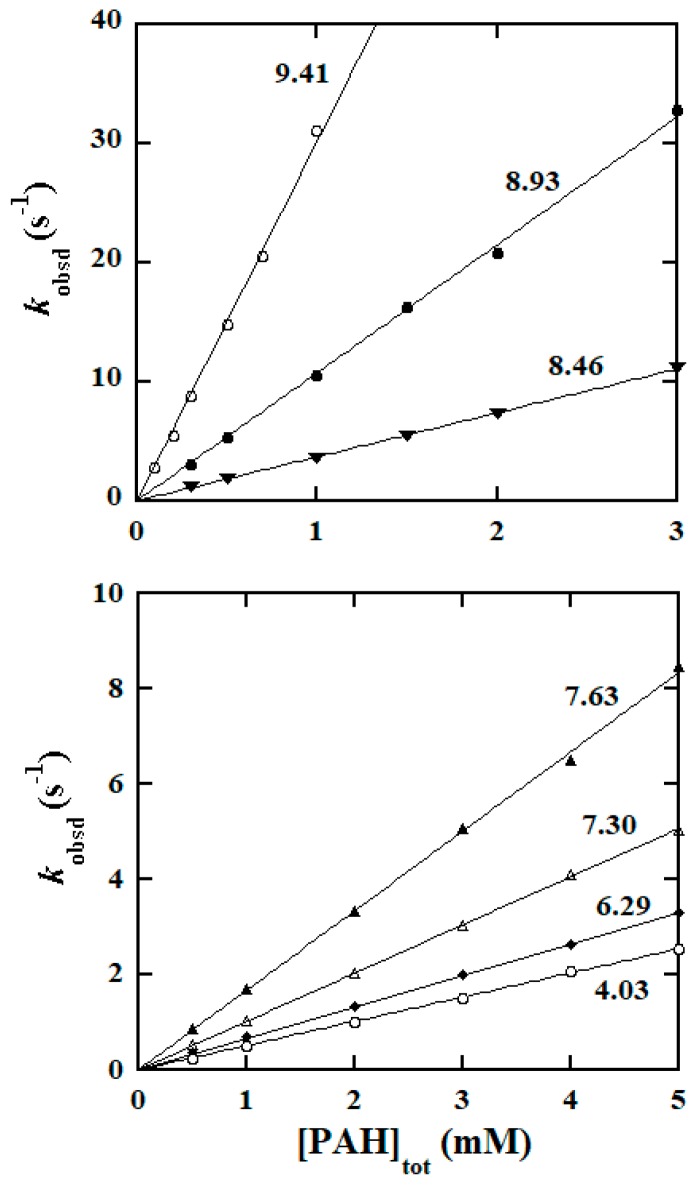
Plots of *k*_obsd_ versus [PAH]_tot_ (phenylacetic hydrazide) at 25.0 °C and different pHs with the numbers around the lines indicating the pH values.

To find the reaction order in [BH]_tot_/[PAH]_tot_ (the subscript tot represents the total concentrations), the effects of varying [BH]_tot_/[PAH]_tot_ on the oxidation rates were investigated in each of an extended series of reaction media. However, the variation of [BH]_tot_/[PAH]_tot_ in each medium was controlled to not induce any pH changes in that particular medium. Plots of *k*_obsd_ versus [Hydrazide]_tot_ are illustrated in Figure 2 in the case of BH and in Figure 3 for the reaction of PAH. No doubt, these plots are linear and passing through the origin, indicating that the oxidation reactions are also first order in [Hydrazide]_tot_. Hence an empirical rate law (expressed by Equation (2)) is established, where *k*′ represents the observed second-order rate constant and *k*_obsd_ = *k*′[Hydrazide]_tot_.

−d[IrCl_6_^2−^]/dt = *k*_obsd_[IrCl_6_^2−^] = *k*′[Hydrazide]_tot_[IrCl_6_^2−^] (2)

**Figure 4:**
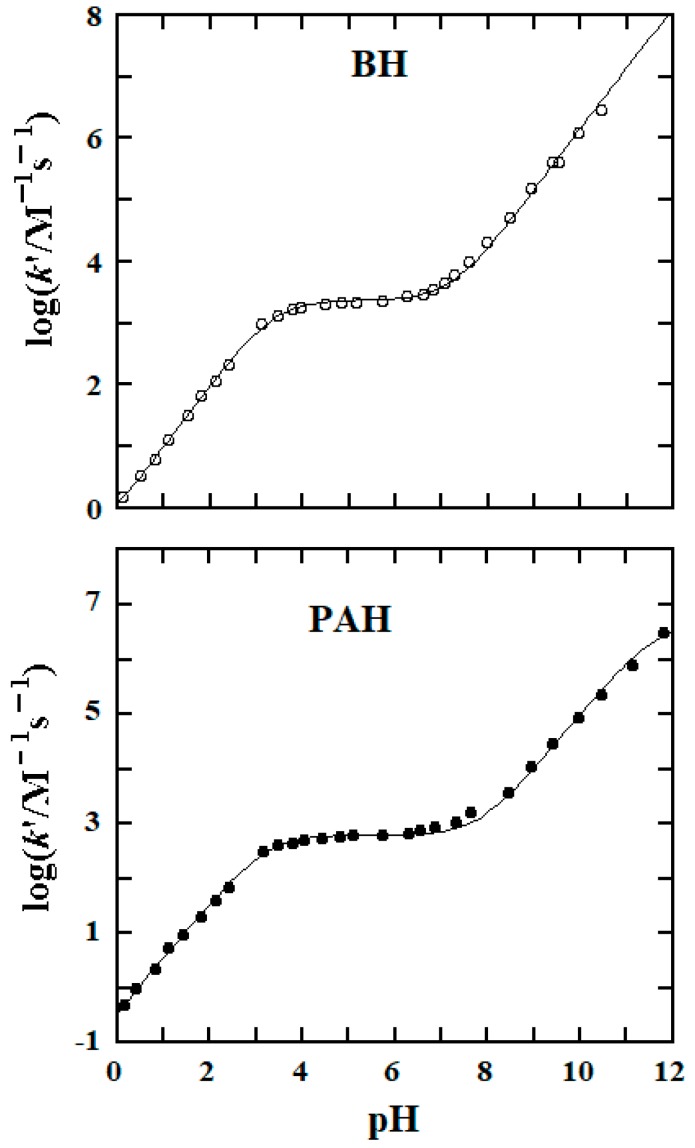
The log*k*′ versus pH profiles at 25.0 °C plotted from the data points for the oxidations of BH and PAH by [IrCl_6_]^2−^ (data points from Table S1 in Supplementary Materials). The solid curves were generated from the best fits of Equation (5) to the experimental data by a weighted nonlinear least-squares simulation.

The oxidation reaction of BH was investigated in a region of 0.11 ≤ pH ≤ 10.46; when pH > 10.5, the reaction became too fast to follow even by the stopped-flow technique. In the case of PAH, the reaction was investigated in a wider pH region (0.16 ≤ pH ≤ 11.78) since the oxidation reaction of PAH was slower than that of BH. Values of *k*′ were computed from the linear plots of *k*_obsd_ versus [Hydrazide]_tot_ at various pHs which were collected from a large amount of data, and are summarized in Table S1 in SM. More visually, the plots of log*k*′ versus pH are given in Figure 4 (data points).

### 2.3. Evaluation of the Reaction Stoichiometry

Spectrophotometric titration was proved to be a good method for determinations of reaction stoichiometries [14,28,29]; it was thus used in the present reaction systems (cf. the experimental section below). From the spectrophotometric titration data, plots of the measured absorbance at 488 nm as a function of [BH]_tot_ are given in Figures S3 and S4 in SM whereas similar plots for the PAH reaction are displayed in Figures S5 and S6 in SM. Clearly, in each of the four figures, all the data points could be traced by two crossing straight lines; from the intersection, a stoichiometric ratio ∆[Ir(IV)]:∆[Hydrazide]_tot_ was estimated. The estimated ratios are summarized in Table 1. Obviously, the ratios in Table 1 point to a clean stoichiometry of ∆[IrCl_6_^2−^]:∆[Hydrazide]_tot_ = 4:1. The same reaction stoichiometry was also derived previously for the oxidations of INH and hydrazines [14,28]. The stoichiometric reaction in the present cases can be described by Equation (3), where *n* = 0 for BH and *n* = 1 for PAH [14].

C_6_H_5_-(CH_2_)_n_-CONHNH_2_ + 4[IrCl_6_]^2−^ + H_2_O → C_6_H_5_-(CH_2_)_n_-COOH + 4[IrCl_6_]^3−^ + 4H^+^ + N_2_ (3)

### 2.4. Product Analysis

**Figure 5:**
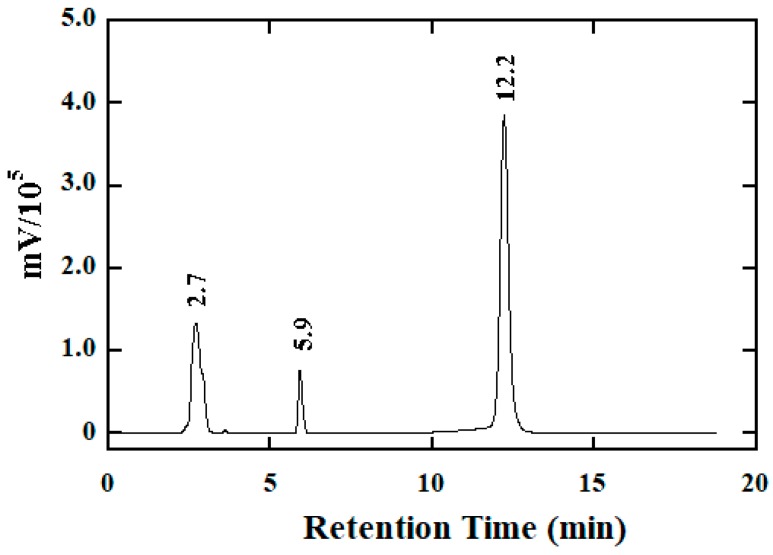
HPLC chromatogram obtained for a reaction mixture containing 2.5 mM [IrCl_6_]^2−^ and 2.5 mM BH in a phosphate buffer of pH 6.34 after a reaction time of 10 min. Peak assignments: Peak at 2.7 min for [IrCl_6_]^3−^; Peak at 5.9 min for benzoic acid; Peak at 12.2 min for BH. Chromatographic conditions: a solvent mixture of H_2_O:MeOH = 4:1 (*v*/*v*) was the mobile phase; the UV detector was set at 261 nm; the flow rate was set at 1.0 mL/min.

To confirm the oxidation products as inferred by Equation (3), RP-HPLC was employed for the analysis of the reaction of BH; Figure 5 displays the chromatogram acquired for a reaction mixture of 2.5 mM BH and 2.5 mM [IrCl_6_]^2−^ in a phosphate buffer of pH 6.34 after a reaction time of about 10 min. In the figure, the peaks were assigned according to the retention times which were identical to those from authentic samples. Moreover, no late peaks in the chromatogram were eluted by an extended elution time. Hence, benzoic acid was identified as the oxidation product of BH.

**Figure 6:**
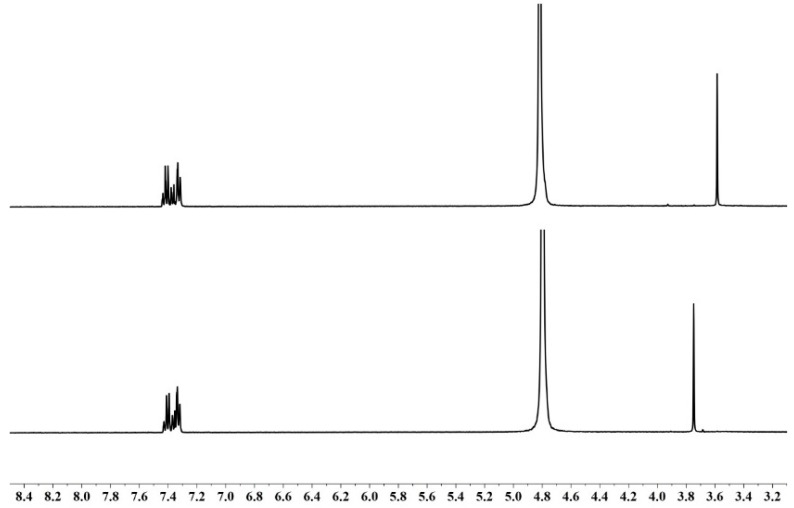
^1^H-NMR spectra recorded on a 400-MHz NMR spectrometer. (**Top**): 1 mM PAH in D_2_O. Assignments of chemical shift for C_6_H_5_-CH_2_-CONHNH_2_: -C**H_2_**-, *δ* 3.59 (s); C_6_**H_5_**-, *δ* 7.32–7.44 (m). (**Bottom**): A reaction mixture of 1 mM PAH and 5 mM [IrCl_6_]^2−^ in D_2_O after a reaction time of 5 h. Assignments of chemical shift for C_6_H_5_-CH_2_-COOH: -C**H_2_**-, *δ* 3.75 (s); C_6_**H_5_**-, *δ* 7.32–7.43 (m).

For the oxidation of PAH by Ir(IV), the ^1^H-NMR spectra acquired are shown in Figure 6, together with the assignments of NMR signals; 3-(trimethylsilyl)propionic acid-*d*_4_ sodium salt (TSP) was utilized as the reference of the NMR shifts in the spectra. The spectra indicate that when more than a stoichiometric amount of Ir(IV) was used in the reaction mixture, all the reactant PAH was cleanly oxidized to phenylacetic acid. It was observed that the excess of Ir(IV) could not oxidize phenylacetic acid that was produced from the oxidation reaction; this was not surprising since Ir(IV) did not oxidize the HAc–NaAc buffers. Phenylacetic acid was thus confirmed as the oxidation product of PAH, justifying Equation (3).

## 3. Discussion

### 3.1. Mechanistic Analysis

**Figure 7:**
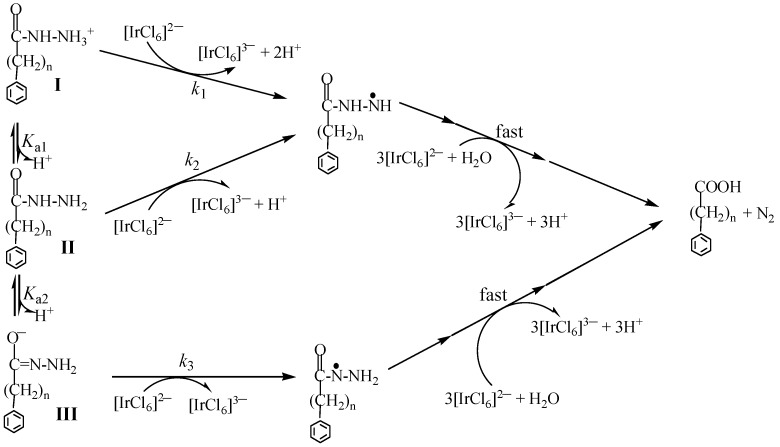
A reaction mechanism proposed for the oxidations of BH (*n* = 0) and PAH (*n* = 1) by [IrCl6]^2−^ in which the reactions described by *k*_1_–*k*_3_ are the rate-determining steps.

For BH and PAH in aqueous solution in the present work, three protolysis species (**I**–**III** shown in Figure 7) are involved across the wide pH range used in present work [14]. The elucidated kinetic characters for the present reaction systems (such as well-defined second-order kinetics, rapid scan spectra, the reaction stoichiometry and the oxidation products) echo those revealed in the INH-Ir(IV) reaction system [14]. Moreover, even the shape of log*k*′ versus pH profiles in Figure 4 is also similar to that obtained for the INH-Ir(IV) reaction. By analog, a reaction mechanism portrayed in Figure 7 is suggested for the present reaction systems in which the reactions denoted by *k*_1_–*k*_3_ are the rate-determining steps. Two types of hydrazyl free radicals (species **IV** and **V**) were inferred to be generated in the rate-determining steps [11,12,13,14,26,27], and were followed by three consecutive and fast reactions, leading to formation of benzoic acid/phenylacetic acid [14].

Rate expression in Equation (4) was attained according to the reaction mechanism in Figure 7, where a_H_ represents the proton activity which corresponds exactly to the pH measurements.

4(*k*_1_a_H_^2^ + *k*_2_*K*_a1_a_H_ + *k*_3_*K*_a1_*K*_a2_) −d[IrCl_6_^2−^]/dt = ^_______________________________________^[Hydrazide]_tot_[IrCl_6_^2−^] a_H_^2^ + *K*_a1_a_H_ + *K*_a1_*K*_a2_ (4)

Equation (4) conforms to the empirical Equation (2), rendering:

4(*k*_1_a_H_^2^ + *k*_2_*K*_a1_a_H_ + *k*_3_*K*_a1_*K*_a2_) *k*′ = ^_______________________________________^ a_H_^2^ + *K*_a1_a_H_ + *K*_a1_*K*_a2_ (5)

### 3.2. pK_a_ Values and Rate Constants of the Rate-Determining Steps

Determination of p*K*_a_ values for hydrazines from the well-defined kinetic data offered a good approach for the Ir(IV)-hydrazine reaction systems [28]; this was based on the measured kinetic data in the pH ranges covering the p*K*_a_ values of hydrazines. For the present reaction systems, the protolysis constants *K*_a1_ and *K*_a2_ of BH and PAH at 25.0 °C and *μ* = 1.0 M have not been reported in the literature. The pH range studied for the PAH reaction was from 0.16 pH 11.78, probably covering both p*K*_a1_ and p*K*_a2_ of PAH, and consequently enabling us to derive these p*K*_a_ values from our kinetic data. Equation (5) was then utilized to simulate the *k*′-pH dependence data by use of a weighted nonlinear least-squares method; in the simulation, *k*_1_, *k*_2_, *k*_3_, *K*_a1_ and *K*_a2_ were all treated as tunable parameters. The simulation provided with a good fit shown in the bottom part of Figure 4, conferring simultaneously the values for these parameters (listed in Table 2). The value of p*K*_a2_ = 11.7 ± 0.2 obtained from the simulation is indeed within the pH region studied kinetically.

**Figure 8:**
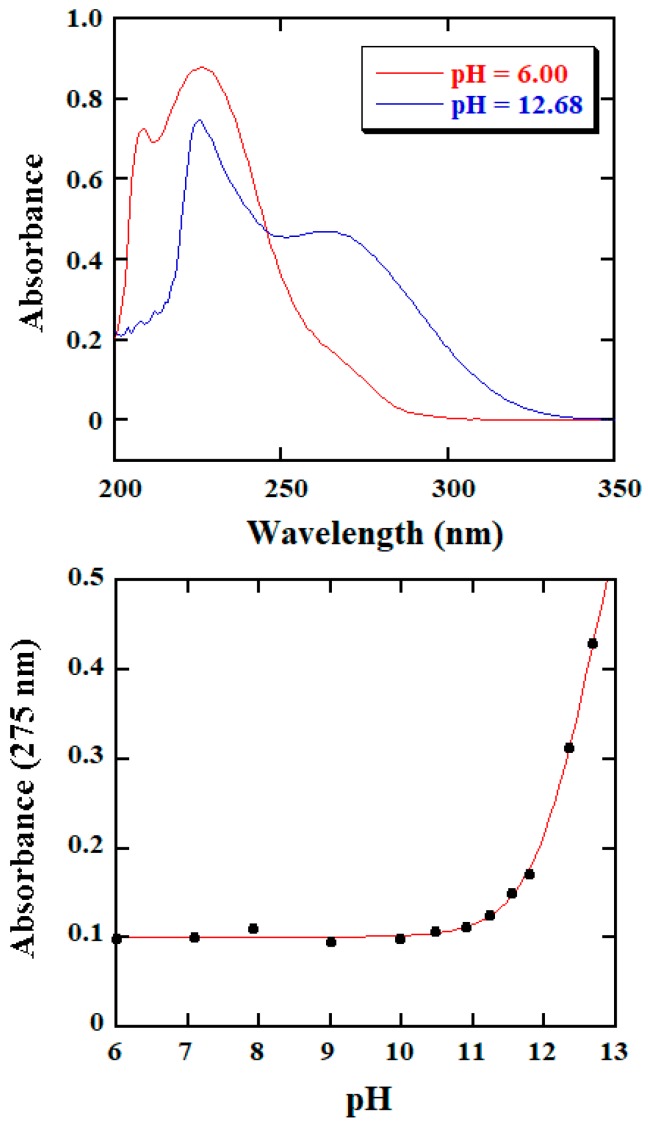
(**Top**): UV-vis spectra of 0.10 mM BH in two buffers of different pH at 25.0 °C and *µ* = 1.0 M. (**Bottom**): Absorbance at 275 nm of 0.10 mM BH solutions as function of pH at 25.0 °C and *µ* = 1.0 M. The solid curve was obtained from the best fit of Equation (6) to the experimental data using a nonlinear least-squares method.

The p*K*_a1_ value of BH is expected to be between 3 and 4 [30], which is in the pH region studied kinetically in this work while that of p*K*_a2_ is anticipated to be >12 at 25.0 °C and *μ* = 1.0 M [30], being beyond the pH region of the kinetic data collection. We thus determined the p*K*_a2_ value of BH spectrophotometrically [31]. The UV-vis spectra recorded for 0.10 mM BH in the buffer solutions of pH 6.00 and 12.68 are given in the top part of Figure 8, where the spectra originate predominantly from species **II** and **III** of BH, respectively. The wavelength of 275 nm was then employed for the measurements of absorption values.

A series of BH solutions with varied pH buffers were prepared in which [BH]_tot_ = 0.10 mM was kept constant; absorption values at 275 nm were measured after those solutions were equilibrated at 25.0 °C for 10 min. The measured value as a function of pH is given in the bottom part of Figure 8 (data points).

Abs (275 nm) = [BH]_tot_{*ε*_3_ + *ε*_2_·10^(p*K*^_a2_^− pH)^}/{1 + 10^(p*K*^_a2_^− pH)^} (6)

Equation (6) was then employed to simulate the data [31], using a nonlinear squares method, where*ε*_2_ and*ε*_3_ represent the molar absorptivities of species **II** and **III**, respectively. The simulation resulted in a good fit, generating the values of*ε*_2_ = (1.00 ± 0.02) × 10^3^ M^−1^cm^−1^, *ε*_3_ = (7.3 ± 0.4) × 10^3^ M^−1^cm^−1^, and p*K*_a2_ = 12.6 ± 0.1 at 25.0 °C and *μ* = 1.0 M.

Equation (5) was then used to simulate the *k*′–pH dependence data for the BH reaction; in the simulation, *k*_1_, *k*_2_, *k*_3_, and *K*_a1_ were treated as adjustable parameters and the value of *K*_a2_ obtained above was used as a direct input. The simulation provided an excellent fit shown in the top part of Figure 4 whereas the acquired values of *k*_1_, *k*_2_, *k*_3_, and *K*_a1_ are listed in Table 2.

### 3.3. Probing the Activation Process

After evaluation of the protolysis constants and the rate constants of the rate-determining steps, we were able to create species of BH/PAH versus pH distribution diagrams (the top parts of Figures S7 and S8 in SM) and the reactivity of BH/PAH species versus pH distribution diagrams (bottom parts of Figures S7 and S8 in SM) [14]. Figures S7 and S8 demonstrate that species **II** of BH/PAH (cf. Figure 7) contributes predominantly in both distributions between pH 5 and 6, which corresponds to the plateau regions in the log*k*′ versus pH plots. In this small region, Equation (5) can be simplified to *k*′ ≈ 4*k*_2_ (or *k*_2_ ≈ *k*′/4). The oxidation reactions were thus investigated at several temperatures in this region; the results are summarized in Figures S9 and S10 in SM (the top parts) and in Table 3. The Eyring plots for *k*_2_ are displayed in the bottom parts of Figures S9 and S10 for the reactions of BH and PAH. Activation parameters were calculated from these plots and are also listed in Table 3. Activation entropies ∆*S*_2_^‡^ = −72 J∙K^−1^∙mol^−1^ for BH and ∆*S*_2_^‡^ = −66 J∙K^−1^∙mol^−1^ for PAH are very close to each other and are of the middle-ranged negative values. These values, reflecting the activation processes between [IrCl_6_]^2−^ and the neutral forms of BH and PAH, are consistent favorably with the nature of the second-order kinetics, where a compact structure of the transition state is expected. When the salient features of the rapid scan spectra are put together with the middle-ranged negative values of activation entropies, an outer-sphere electron transfer likely took place in the rate-determining step denoted by *k*_2_ [14,28]. The same mode of electron transfer is expected to occur for the reactions expressed by *k*_1_ and *k*_3_ although it was not possible to determine the activation parameters for these reactions.

### 3.4. Comparison of the Rate Constants

For the oxidations of aryl hydrazides by [IrCl_6_]^2−^, the most surprising observation was that the reactivities of the protolysis species of hydrazides vary by about nine orders of magnitude [14]. This huge reactivity difference is also observed in the oxidation reaction of BH by [IrCl_6_]^2−^, being as *k*_1_:*k*_2_:*k*_3_ = 1:1.3 × 10^4^:3.2 × 10^9^. BH is very close to INH in structure and the reactivities of its neutral and enolate forms are about the same as those of INH. INH is a frontline anti-tubercular drug but BH has essentially no anti-tubercular activity. Thus, the vital role played by the pyridine nitrogen in INH is not related directly to their reactivity in the reduction of a single electron oxidant. For the PAH–[IrCl_6_]^2−^ reaction, the ratio of *k*_2_:*k*_3_ = 1:7.6 × 10^3^ becomes smaller but is still large. Thus, it can be concluded that the enolate forms of aryl hydrazides are exceptionally reactive towards reduction of [IrCl_6_]^2−^. This exceptionally high reactivity makes it possible that aryl hydrazides are potentially good candidates for antioxidants [32]. This also accounts for the good chemical preserving properties of hydrazides [2]. Another surprising observation in this work is that a methylene group reduces the reactivity of the enolate form of PAH about 120 times from that of BH.

### 3.5. Structure–Reactivity Relationship

**Figure 9:**
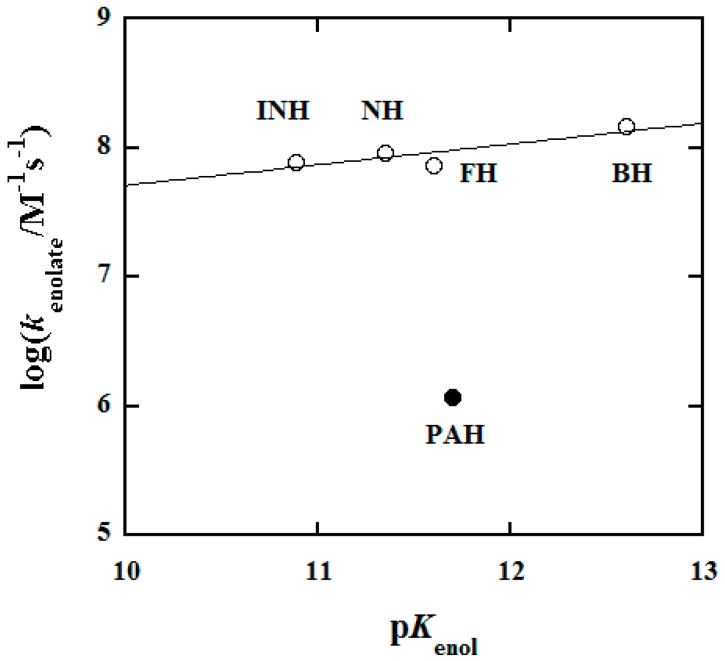
Plot of log*k*_enolate_ versus p*K*_enol_ in the oxidation reactions of hydrazides by [IrCl_6_]^2−^ in aqueous solution at 25.0 °C and *µ* = 1.0 M. INH, NH, FH, and BH are aryl hydrazides.

Table 4 summarizes the main results acquired so far for the oxidations of hydrazides by [IrCl_6_]^2−^ including a very recent one (2-furoic hydrazide (FH), cf. Figure S1 in SM) studied by the Shi group [33]. The p*K*_a_ values for the deprotonation of R-CONHNH_3_^+^ locate in a small region, only varying from 3.04 to 3.67 whereas the reactivity of the neutral forms of hydrazides changes from 157 to 1120 M^−1^s^−1^. Clearly, there is no correlation between the reactivity and p*K*_a_ values (Table 4), which supports the conclusion drawn in the oxidations of hydrazine and substituted hydrazines by [IrCl_6_]^2−^ [28]. On the other hand, a linear correlation is found between the reactivity of the enolate forms of the aryl hydrazides (INH, NH, FH, and BH) and the p*K*_enol_ values as shown in Figure 9; the correlation is expressed by: log*k*_enolate_ = (0.16 ± 0.07)p*K*_enol_ + (6.1 ± 0.8). The big intercept and the small slope of the correlation indicate that the intrinsic reactivities of the enolate forms of aryl hydrazides are very high but are not very sensitive to the enolate basicities. These characters may pave a way for a rational design of new antioxidants based on aryl hydrazides. PAH no longer being an aryl hydrazide drops the correlation, suggesting that other aliphatic hydrazides may fall off the correlation as well. Hence, more data are needed to test whether aliphatic hydrazides together with PAH will follow another correlation.

## 4. Materials and Methods

### 4.1. Chemicals

Benz(o)hydrazide (BH, 98%), phenylacetic hydrazide (PAH, 98%), benzoic acid, sodium hexachloroiridate(IV) hexahydrate (Na_2_IrCl_6_·6H_2_O, 99.9%), and 2,4-pyridinedicarboxylic acid (PDCA, ≥98%) were purchased from Sigma–Aldrich (Sigma Shanghai Branch, China). The purity of BH and PAH was checked by RP-HPLC and ^1^H-NMR spectroscopy, respectively, confirming a good purity. Acetic acid, sodium acetate, sodium dihydrogen phosphate, disodium hydrogen phosphate, trisodium phosphate, sodium bicarbonate, sodium carbonate, sodium chloride, hydrochloric acid, perchloric acid, sodium perchlorate, D_2_O and TSP were obtained either from Alfa Aesar or from Fisher Scientific (Alfa Aesar Shanghai Branch and Fisher Shanghai Branch, China); these chemicals were all of analytical grade and used without further purification. Methanol in HPLC grade and standard buffers of pH 4.00, 7.00, and 10.00 were also obtained from Fisher Scientific. Doubly distilled water was utilized to prepare all the solutions.

### 4.2. Buffers and Reaction Media

The following buffering pairs of AcOH/NaOAc, NaH_2_PO_4_/Na_2_HPO_4_, NaHCO_3_/Na_2_CO_3_, and Na_2_HPO_4_/Na_3_PO_4_ (all about 0.2 M) were combined to cover the pH range from 3.15 to 12.68; all the buffers which contained 2 mM PDCA [19] were adjusted to an ionic strength (*μ*) of 1.0 M by use of NaClO_4_. The pH values were measured immediately before use with an Accumet Basic AB150 Plus pH meter equipped with an Accumet combination pH electrode (Fisher Scientific, Pittsburgh, PA, USA); the electrode was calibrated by use of the standard buffers of pH 4.00, 7.00, and 10.00 before the pH measurements. The acidic reaction media were prepared by use of the combinations of 1.00 M HClO_4_ and 1.00 M NaClO_4_ solutions, where [H^+^] = [HClO_4_]. For these media, their pH values were estimated by: pH = −log[H^+^] + 0.11, which is based on a mean activity coefficient of 0.77 in solutions of 1.00 M NaClO_4_ [34].

### 4.3. Stoichiometric Investigation

The reactions were investigated in two reaction media: a phosphate buffer of pH 6.31 and an acidic medium of [H^+^] = 0.010 M. A series of solution mixtures were prepared in which [IrCl_6_^2^^−^] = 0.40 mM was remained constant and [Hydrazine]_tot_ was varied from 0 to 0.40 mM in each of the two media, where [Hydrazide]_tot_ = [BH]_tot_/[PAH]_tot_ and the subscript pertains to total concentration. The reaction mixtures were aged for certain times, then the absorbance was measured at 488 nm employing a TU-1950 spectrophotometer (Persee Inc., Beijing, China) and 1.00 cm quartz cells. The spectrophotometer was connected to a water bath circulation from a thermostat (Lauda Alpha RA8, Belran, NJ, USA). The temperature could be controlled to ±0.1 °C (vide infra).

### 4.4. Product Analysis by RP-HPLC and NMR Spectra

For identification of the oxidation product of BH, reaction mixtures of BH with [IrCl_6_]^2−^ were analyzed by reversed-phase high performance liquid chromatography (RP-HPLC) using a Shimadzu LC-20 AD HPLC system equipped with a UV detector (Shimadzu Corporation, Kyoto, Japan). A C18 column of Shimadzu (250 × 4.6 mm, 5 µm in particle size) and an injection loop of 20 µL were used for sample separations and injections. Moreover, the injection loop was always fully filled with samples. After optimizations of mobile phase in an isocratic elution mode, a solvent mixture of H_2_O:MeOH = 4:1 (*v*/*v*) was chosen as the mobile phase. The UV detector was set at 261 nm and the flow rate was at 1.0 mL/min. Under the optimized conditions, a reaction mixture containing 2.5 mM BH and 2.5 mM [IrCl_6_]^2−^ in a phosphate buffer of pH 6.34 after a reaction time of 10 min was subjected to analysis.

In the case of PAH, ^1^H-NMR spectroscopy (AVANCE NEO 400 MHz NMR spectrometer, Bruker, Switzerland) was utilized to analyze the oxidation product. Two samples were prepared for NMR experiments: (a) 1 mM PAH in D_2_O which contained 0.02% TSP and (b) a reaction mixture of 1 mM PAH and 5 mM [IrCl_6_]^2^^−^ after a reaction time of about 5 hrs.

### 4.5. Kinetic Measurements

A stock solution of 1.0 mM [IrCl_6_]^2−^ was prepared and used daily by dissolving the desired amount of Na_2_IrCl_6_·6H_2_O in a solution mixture containing 0.99 M NaClO_4_ and 0.01 M HCl. Stock solutions of BH/PAH were prepared by adding the required amount of BH/PAH into a reaction medium of specific pH and then flushed for 5 min with nitrogen of high purity. For kinetic measurements, solutions of [IrCl_6_]^2−^ and BH/PAH were prepared by dilution of the stock solutions with the same medium and then flushed for about 5 min with the nitrogen. Reactions were initiated by mixing equal volumes of the [IrCl_6_]^2−^ and BH/PAH solutions directly on an SX-20 stopped-flow spectrometer (Applied Photophysics Ltd., Leatherhead, UK); the temperature was also controlled to ±0.1 °C using another thermostat of Lauda Alpha RA8. Moreover, the reaction solutions were only used for a couple of hours. The reactions were investigated under pseudo first-order conditions with [Hydrazide]_tot_ ≥ 10·[IrCl_6_^2−^].

## 5. Conclusions

The oxidation reactions of BH and PAH by [IrCl_6_]^2−^ have been characterized in a wide pH range by use of stopped-flow spectral, rapid spectral scan, RP-HPLC, and NMR spectroscopic techniques. The nature of well-defined second-order kinetics of the reactions warrants the evaluation of the rate constants of rate-determining steps and the protolysis constants for both BH and PAH. This work clearly confirmed the earlier findings made by the Shi group [14] that the enolate forms of aryl hydrazides have exceptionally high reactivities towards reduction of Ir(IV). Moreover, for the aryl hydrazides, a linear correction of log*k*_enolate_ = (0.16 ± 0.07)p*K*_enol_ + (6.1 ± 0.8) is unraveled for the first time. Collectively, this work together with previous investigations [14,33] may pave a way for a rational design of new antioxidants based on aryl hydrazides. Additionally, the present work also offers the p*K*_a_ values for BH and PAH at 25.0 °C and *μ* = 1.0 M.

## Figures and Tables

**Table 1:** Stoichiometric ratios determined for oxidation reactions of BH and PAH by [IrCl_6_]^2−^ at room temperature.

Reductant	∆[Ir(IV)]:∆[Hydrazide]_tot_	Reaction Medium (Reaction Time)
BH	4.0:1.03 ± 0.03	pH 6.31 phosphate buffer (5 min)
	4.0:1.05 ± 0.03	0.010 M HClO_4_ (2 h)
PAH	4.0:0.097 ± 0.03	pH 6.31 phosphate buffer (30 min)
	4.0:1.00 ± 0.03	0.010 M HClO_4_ (2 h)

**Table 2:** Rate constants for the rate-determining steps and protolysis constants in Figure 7 for the reactions of BH and PAH determined at 25.0 °C and ionic strength *μ* = 1.0 M.

Hydrazide	*k*_m_/p*K*_am_	Values
BH	*k*_1_	0.046 ± 0.013 M^−1^s^−1^
	*k*_2_	597 ± 9 M^−1^s^−1^
	*k*_3_	(1.47 ± 0.05) × 10^8^ M^−1^s^−1^
	p*K*_a1_	3.37 ± 0.09
	p*K*_a2_	12.6 ± 0.1
PAH	*k*_1_	0 (or indeterminate)
	*k*_2_	157 ± 5 M^−1^s^−1^
	*k*_3_	(1.19 ± 0.06) × 10^6^ M^−1^s^−1^
	p*K*_a1_	3.24 ± 0.08
	p*K*_a2_	11.7 ± 0.2

**Table 3:** Rate constant *k*_2_ as a function of temperature and activation parameters for oxidation of the neutral forms of BH and PAH defined in Figure 7 by [IrCl_6_]^2−^ at *μ* = 1.0 M.

Hydrazide	*t*/°C	*k*_2_/M^−1^s^−1^	∆*H*_2_^‡^/kJ∙mol^−1^	∆*S*_2_^‡^/J∙K^−1^∙mol^−1^
BH	15.0	297 ± 9	36.2 ± 0.7	−72 ± 5
	20.0	397 ± 15		
	25.0	521 ± 20		
	30.0	654 ± 25		
	35.0	857 ± 27		
PAH	15.0	86 ± 2	40.7 ± 0.9	−66 ± 4
	20.0	119 ± 3		
	25.0	158 ± 4		
	30.0	205 ± 5		
	35.0	283 ± 9		

**Table 4:** A summary of the major results for the oxidations of hydrazides by [IrC_l6_]^2−^ at 25.0 °C and *μ* = 1.0 M.

Hydrazide	p*K*_a_^a^	*k*/M^−1^s^−1 b^	p*K*_enol_^c^	*k*_enolate_/M^−1^s^−1 d^	Ref.
INH	3.67	1.13 × 10^3^	10.89	7.9 × 10^7^	[14]
NH	3.49	261	11.35	9.1 × 10^7^	[14]
FH	3.04	620	11.6	7.3 × 10^7^	[34]
BH	3.37	597	12.6	1.47 × 10^8^	This Work
PAH	3.24	157	11.7	1.19 × 10^6^	This Work

^a^ p*K*_a_ for RCO–NH–NH_3_^+^; ^b^ Second-order rate constant for RCO–NH–NH_2_; ^c^ p*K*_a_ for the enol form of hydrazide; ^d^ The second-order rate constant for oxidation of enolate form of hydrazides by [IrCl_6_]^2−^.

## Supplementary Materials

The following are available online, Table S1: Observed second-order rate constants *k*’ for oxidations of BH and PAH by [IrCl_6_]^2−^ as a function of pH at 25.0 °C and 1.0 M ionic strength. Figure S1: Structures of hydrazides including aryl hydrazides BH, INH, NH, and FH. Figure S2: Kinetic traces acquired from the data points in Figure 1. The solid curves were obtained from the best fits of the experimental data to Equation (1). Figure S3: Spectrophotometric titration: absorbance at 488 nm for a series of reaction mixtures of [IrCl_6_]^2−^ with BH in which [BH]_tot_ was changed from 0 to 0.40 mM and [Ir(IV)] = 0.40 mM was kept constant. Reaction medium: phosphate of pH 6.31 and *μ* = 1.0 M. Reaction time: about 5 min for each of the mixtures at room temperature. Figure S4: Spectrophotometric titration: absorbance at 488 nm for a series of reaction mixtures of [IrCl_6_]^2−^ with BH in which [BH]_tot_ was changed from 0 to 0.40 mM and [Ir(IV)] = 0.40 mM was kept constant. Reaction medium: [H^+^] = 0.010 M, and *μ* = 1.0 M. Reaction time: about 2 h for each of the mixtures at room temperature. Figure S5: Spectrophotometric titration: absorbance at 488 nm for a series of reaction mixtures of [IrCl_6_]^2−^ with PAH in which [PAH]_tot_ was varied from 0 to 0.40 mM and [Ir(IV)] = 0.40 mM was retained constant. Reaction medium: phosphate buffer of pH 6.31 and *μ* = 1.0 M. Reaction time: about 30 min for each of the mixtures at room temperature. Figure S6: Spectrophotometric titration: absorbance at 488 nm for a series of reaction mixtures of [IrCl_6_]^2−^ with PAH in which [PAH]_tot_ was varied from 0 to 0.40 mM and [Ir(IV)] = 0.40 mM was retained constant. Reaction medium: [H^+^] = 0.010 M, and *μ* = 1.0 M. Reaction time: about 2 h for each of the mixtures at room temperature. Figure S7: (Top): BH species versus pH distribution diagram at 25.0 °C and *μ* = 1.0 M, which was calculated by use of p*K*_a1_ = 3.37 and p*K*_a2_ = 12.6 in Table 2. (Bottom): Reactivity versus pH distribution diagram for the BH species in the reduction of [IrCl_6_]^2−^; the above p*K*_a_ values and *k*_1_ = 0.046, *k*_2_ = 597, and *k*_3_ = 1.47 × 10^8^ M^−1^s^−1^ in Table 1 were utilized in the calculations. Species **I**–**III** of BH are described in Figure 7. Figure S8: (Top): PAH species versus pH distribution diagram at 25.0 °C and *μ* = 1.0 M, which was calculated by use of p*K*_a1_ = 3.24 and p*K*_a2_ = 11.7 in Table 2. (Bottom): Reactivity versus pH distribution diagram for the PAH species in the reduction of [IrCl_6_]^2−^; the above p*K*_a_ values and *k*_1_ = 0, *k*_2_ = 157, and *k*_3_ = 1.19 × 10^6^ M^−1^s^−1^ in Table 1 were utilized in the calculations. Species **I**–**III** of PAH are described in Figure 7. Figure S9: (Top): Linear plots of *k*_obsd_ versus [BH]_tot_ in a buffer of pH 5.10. (Bottom): Eyring plot of the rate-determining step described by *k*_2_ in the oxidation of BH by [IrCl_6_]^2−^. Figure S10: (Top): Linear plots of *k*_obsd_ versus [PAH]_tot_ in a buffer of pH 5.74. (Bottom): Eyring plot of the rate-determining step described by *k*_2_ in the oxidation of PAH by [IrCl_6_]^2−^.
