# Supplementary material for: Oxidations of Benzhydrazide and Phenylacetic Hydrazide by Hexachloroiridate(IV): Reaction Mechanism and Structure–Reactivity Relationship
Source: Molecules. 2020 Jan 12;25(2):308. doi: 10.3390/molecules25020308 (PMC7024218; doi:10.3390/molecules25020308)
Supplement: Supplementary file 1 [file molecules-25-00308-s001.pdf]

Supplementary Materials for:

Oxidations of Benzhydrazide and Phenylacetic Hydrazide by Hexachloroiridate(IV): Reaction Mechanism and Structure-Reactivity Relationship

Xiaolai Zhang

College of Chemistry and Chemical Engineering, Shandong University, Jinan 250014, Shandong Province, People's Republic of China

One supporting table (Table S1) and ten supporting figures (Figures S1-S10) are included in Supplementary Materials

Table S1. Observed second-order rate constants k' for oxidations of BH and PAH by $[IrCl_6]^{2-}$ as a function of pH at 25.0 ºC and 1.0 M ionic strength.

Hydrazide	pH	$k'/M^{-1}s^{-1}$
BH	0.11	1.49 ± 0.05
	0.51	3.5 ± 0.1
	0.81	6.3 ± 0.2
	1.11	13.0 ± 0.3
	1.51	32.4 ± 0.8
	1.81	66.3 ± 1.3
	2.11	120 ± 4
	2.41	218 ± 6
	3.12	983 ± 30
	3.45	(1.36 ± 0.04) x 10^3
	3.76	(1.67 ± 0.05) x 10^3
	3.96	(1.85 ± 0.04) x 10^3
	4.48	(2.07 ± 0.05) x 10^3
	4.85	(2.16 ± 0.05) x 10^3
	5.14	(2.17 ± 0.05) x 10^3
	5.70	(2.26 ± 0.05) x 10^3
	6.25	(2.78 ± 0.06) x 10^3
	6.59	(2.99 ± 0.06) x 10^3
	6.82	(3.59 ± 0.07) x 10^3
	7.06	(4.55 ± 0.09) x 10^3
	7.28	(6.02 ± 0.12) x 10^3
	7.58	(1.02 ± 0.03) x 10^4
	7.98	(2.09 ± 0.03) x 10^4
	8.49	(5.06 ± 0.08) x 10^4
	8.93	(1.52 ± 0.04) x 10^5
	9.41	(4.08 ± 0.08) x 10^5
	9.53	(4.23 ± 0.08) x 10^5
	9.95	(1.26 ± 0.02) x 10^6
	10.46	(2.9 ± 0.1) x 10^6
PAH	0.16	0.50 ± 0.01
	0.41	1.01 ± 0.02
	0.81	2.30 ± 0.05
	1.11	5.5 ± 0.1

1.41	9.3 ± 0.2
1.81	20.8 ± 0.5
2.11	39.8 ± 0.9
2.41	71.3 ± 2.5
3.15	313 ± 6
3.48	411 ± 9
3.77	458 ± 9
4.03	516 ± 6
4.43	537 ± 7
4.82	580 ± 8
5.09	613 ± 9
5.74	630 ± 9
6.29	652 ± 9
6.55	768 ± 15
6.84	864 ± 16
7.30	$(1.06 \pm 0.02) \times 10^3$
7.63	$(1.67 \pm 0.04) \times 10^3$
8.46	$(3.73 \pm 0.07) \times 10^3$
8.93	$(1.09 \pm 0.02) \times 10^4$
9.41	$(3.02 \pm 0.06) \times 10^4$
9.95	$(8.9 \pm 0.2) \times 10^4$
10.46	$(2.25 \pm 0.04) \times 10^5$
11.12	$(8.0 \pm 0.2) \times 10^5$
11.78	$(3.10 \pm 0.06) \times 10^6$

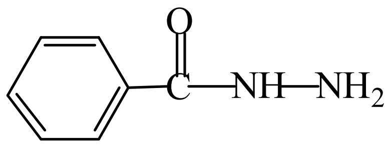

Benz(o)hydrazide (BH)

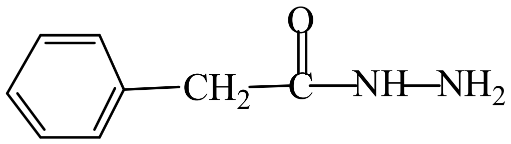

Phenylacetic hydrazide (PAH)

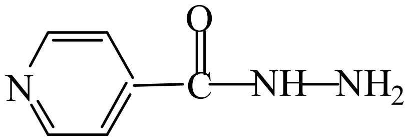

Isoniazid (Isonicotinic hydrazide, INH)

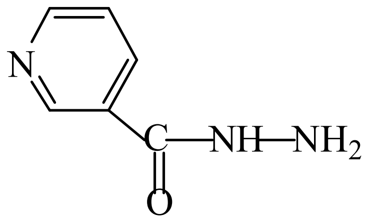

Nicotinic hydrazide (NH)

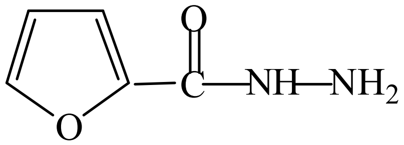

2-Furoic hydrazide (FH)

Figure S1. Structures of hydrazides including aryl hydrazides BH, INH, NH and FH.

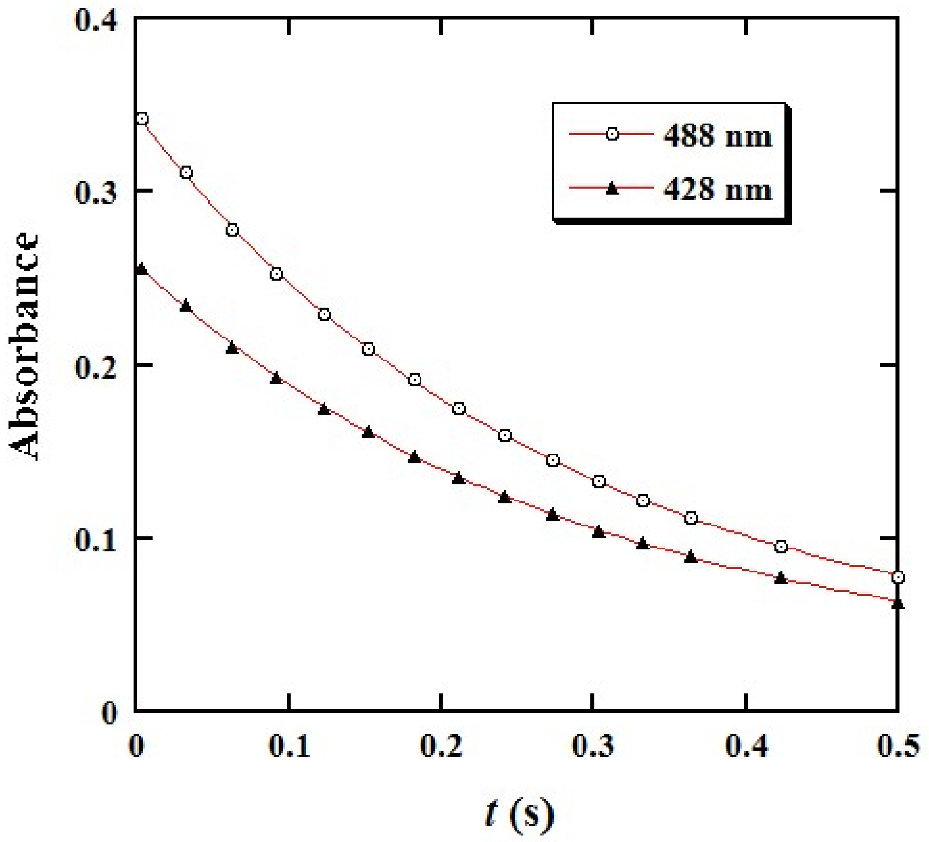

Figure S2. Kinetic traces acquired from the data points in Figure 1 in the text. The solid curves were obtained from the best fits of the experimental data to Equation (1).

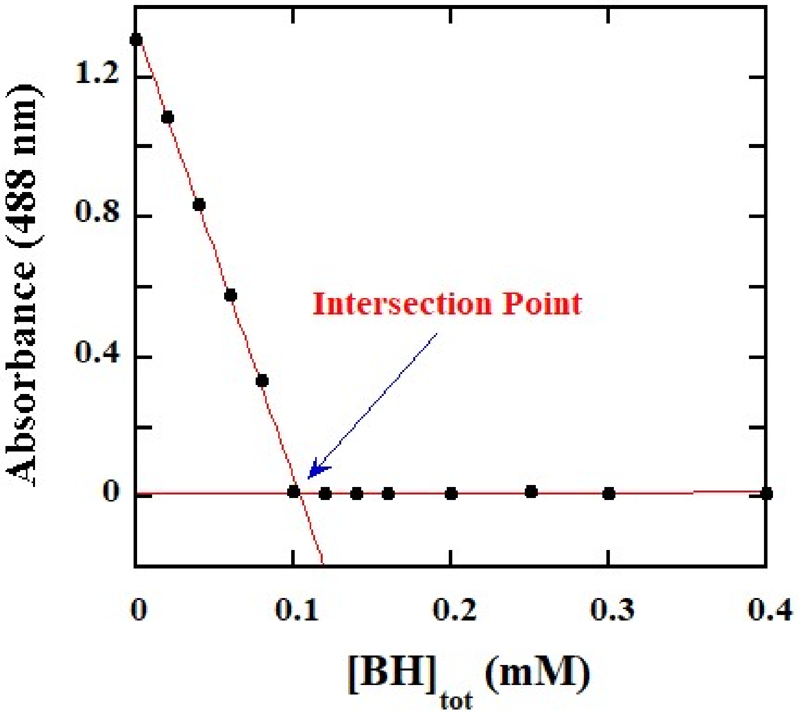

Figure S3. Spectrophotometric titration: absorbance at 488 nm for a series of reaction mixtures of $[IrCl_6]^{2-}$ with BH in which $[BH]_{tot}$ was changed from 0 to 0.40 mM and [Ir(IV)] = 0.40 mM was kept constant. Reaction medium: phosphate of pH 6.31 and μ = 1.0 M. Reaction time: about 5 min for each of the mixtures at room temperature.

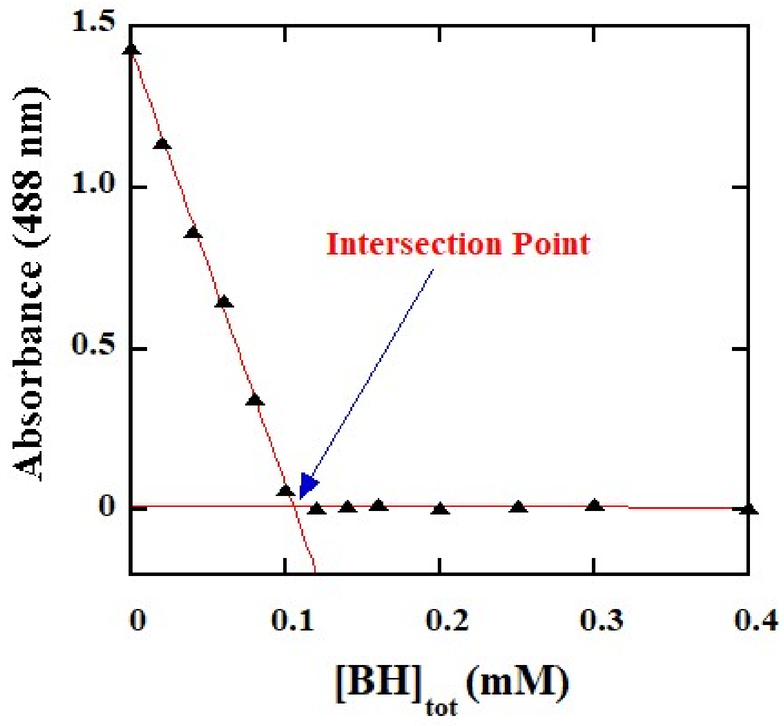

Figure S4. Spectrophotometric titration: absorbance at 488 nm for a series of reaction mixtures of $[IrCl_6]^{2-}$ with BH in which $[BH]_{tot}$ was changed from 0 to 0.40 mM and [Ir(IV)] = 0.40 mM was kept constant. Reaction medium: $[H^+]$ = 0.010 M, and μ = 1.0 M. Reaction time: about 2 h for each of the mixtures at room temperature.

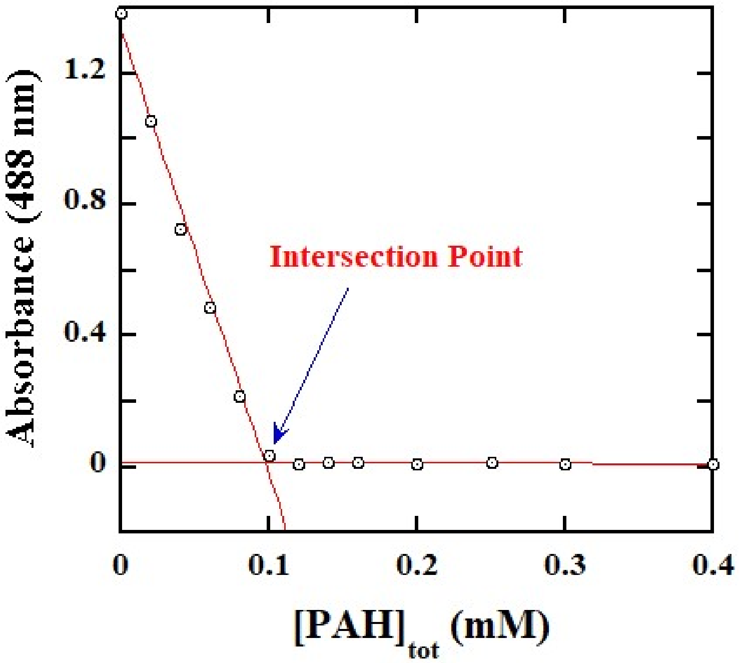

Figure S5. Spectrophotometric titration: absorbance at 488 nm for a series of reaction mixtures of $[IrCl_6]^{2-}$ with PAH in which $[PAH]_{tot}$ was varied from 0 to 0.40 mM and [Ir(IV)] = 0.40 mM was retained constant. Reaction medium: phosphate buffer of pH 6.31 and μ = 1.0 M. Reaction time: about 30 min for each of the mixtures at room temperature.

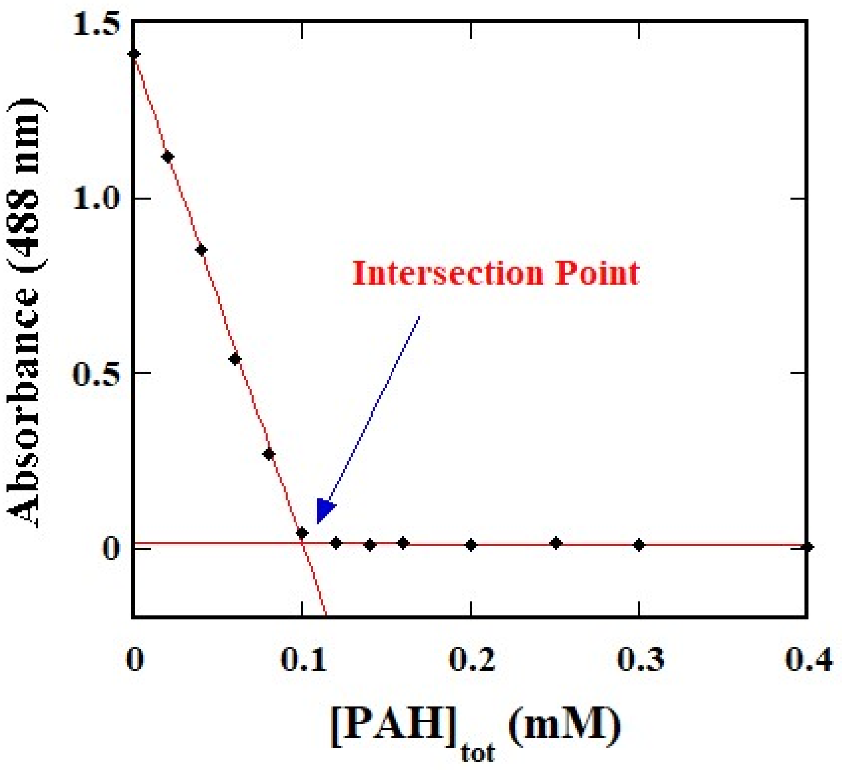

Figure S6. Spectrophotometric titration: absorbance at 488 nm for a series of reaction mixtures of $[IrCl_6]^{2-}$ with PAH in which $[PAH]_{tot}$ was varied from 0 to 0.40 mM and [Ir(IV)] = 0.40 mM was retained constant. Reaction medium: $[H^+]$ = 0.010 M, and μ = 1.0 M. Reaction time: about 2 h for each of the mixtures at room temperature.

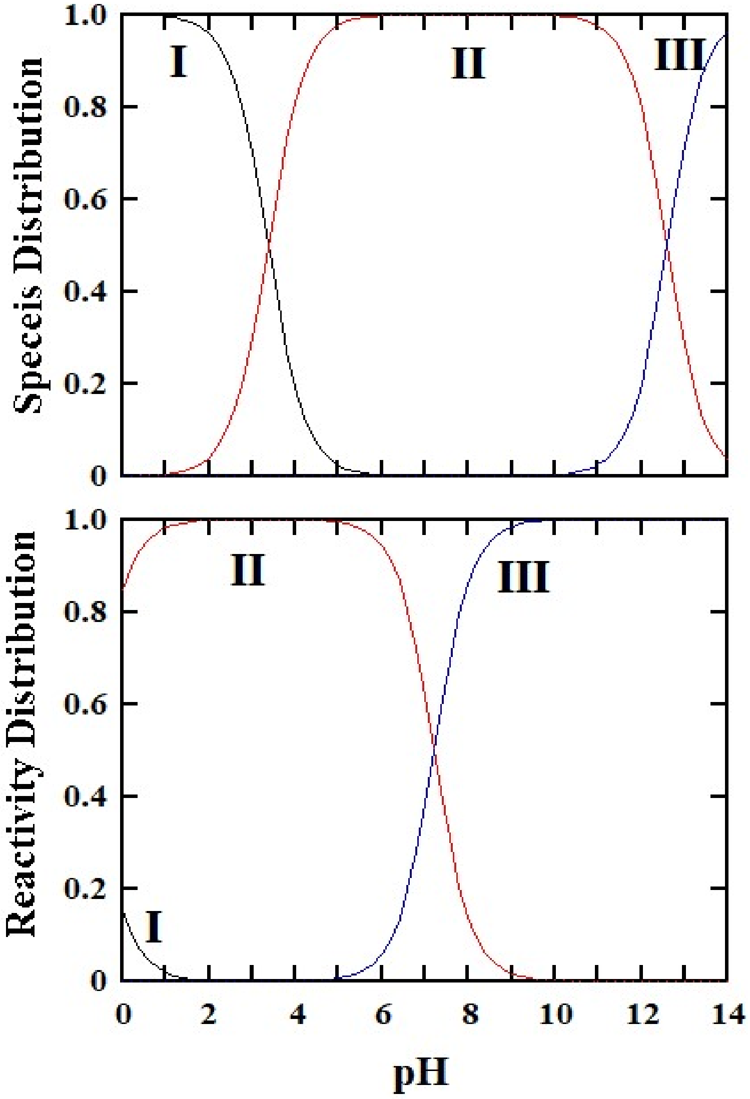

Figure S7. (**Top**): BH species *versus* pH distribution diagram at 25.0 ºC and $\mu = 1.0$ M, which was calculated by use of $pK_{a1} = 3.37$ and $pK_{a2} = 12.6$ in Table 2. (**Bottom**): Reactivity *versus* pH distribution diagram for the BH species in the reduction of $[IrCl_6]^{2-}$; the above pK_a values and $k_1 = 0.046$, $k_2 = 597$, and $k_3 = 1.47 \times 10^8$ $M^{-1}s^{-1}$ in Table 1 were utilized in the calculations. Species **I** – **III** of BH are described in Figure 7.

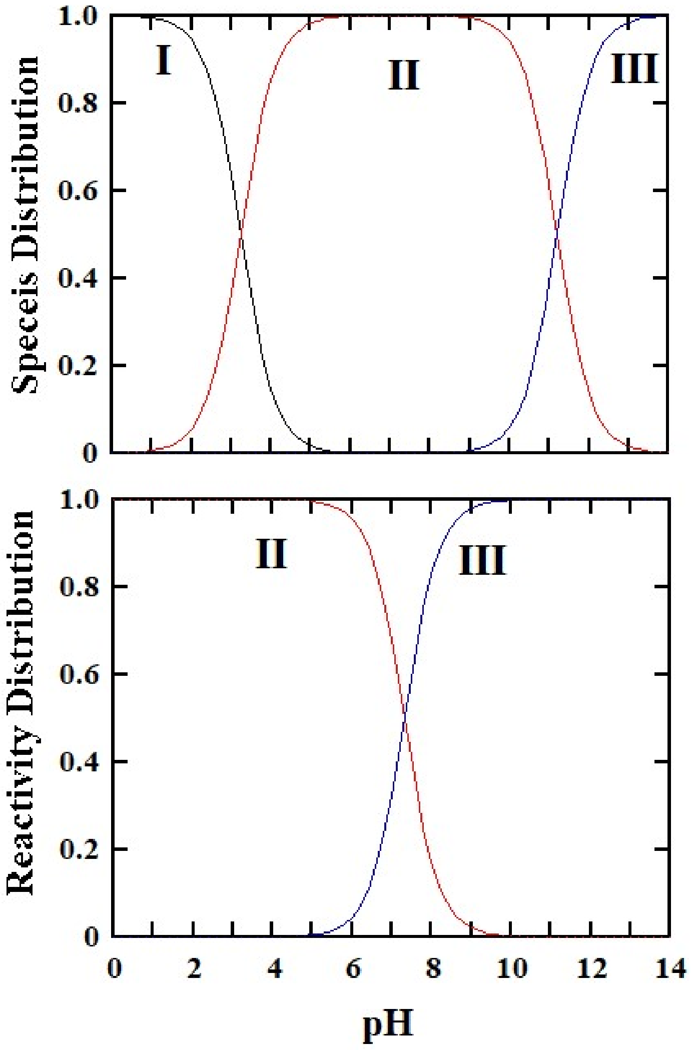

Figure S8. (**Top**): PAH species *versus* pH distribution diagram at 25.0 °C and μ = 1.0 M, which was calculated by use of pK_{a1} = 3.24 and pK_{a2} = 11.7 in Table 2. (**Bottom**): Reactivity *versus* pH distribution diagram for the PAH species in the reduction of $[IrCl_6]^{2-}$; the above pK_a values and k_1 = 0, k_2 = 157, and k_3 = 1.19 x 10^6 $M^{-1}s^{-1}$ in Table 1 were utilized in the calculations. Species **I** – **III** of PAH are described in Figure 7.

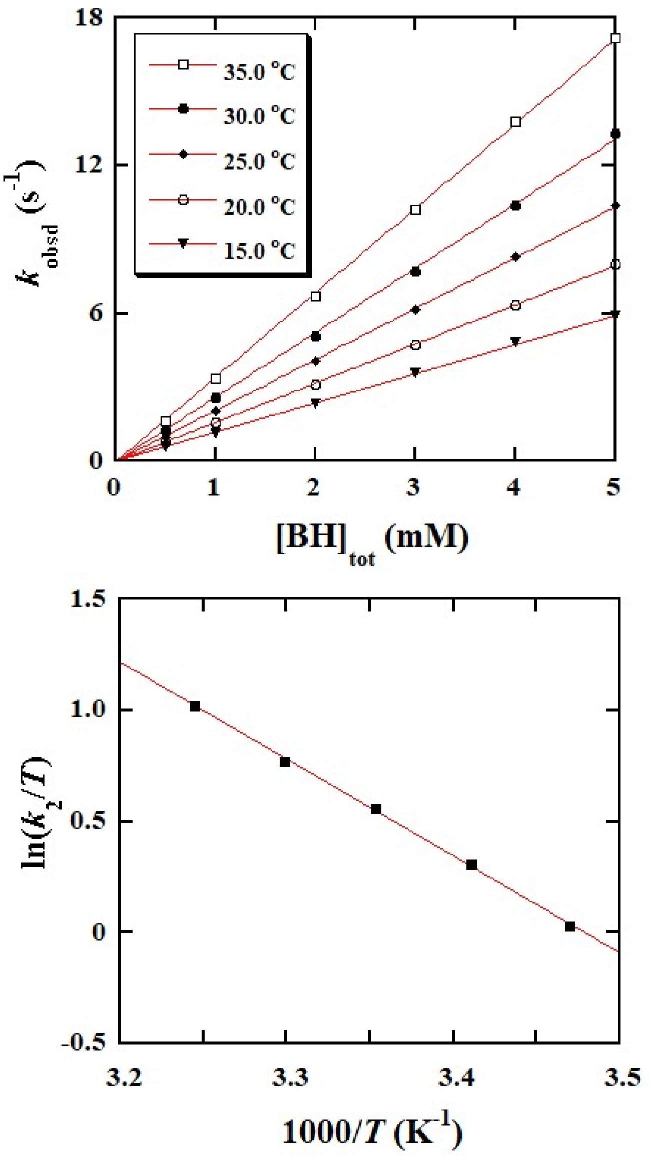

Figure S9. (Top): Linear plots of k_{obsd} *versus* $[BH]_{tot}$ in a buffer of pH 5.10. **(Bottom):** Eyring plot of the rate-determining step described by k_2 in the oxidation of BH by $[IrCl_6]^{2-}$.

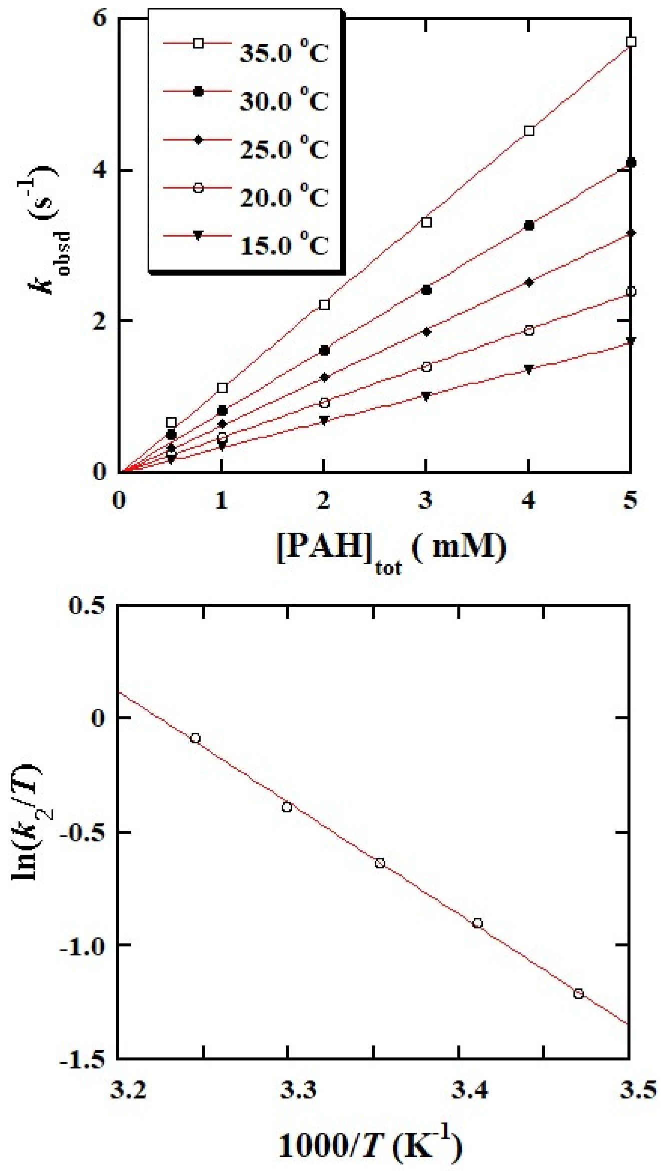

Figure S10. (Top): Linear plots of k_{obsd} *versus* $[PAH]_{tot}$ in a buffer of pH 5.74. **(Bottom):** Eyring plot of the rate-determining step described by k_2 in the oxidation of PAH by $[IrCl_6]^{2-}$.
